# A New Black Elderberry Dye Enriched in Antioxidants Designed for Healthy Sweets Production

**DOI:** 10.3390/antiox8080257

**Published:** 2019-07-31

**Authors:** Dżugan Małgorzata, Pizoń Aleksandra, Tomczyk Monika, Kapusta Ireneusz

**Affiliations:** 1 Department of Chemistry and Food Toxicology, Faculty of Biology and Agriculture, University of Rzeszów, Ćwiklińskiej 1a St., 35-601 Rzeszów, Poland; 2 Department of Food Technology and Human Nutrition, Faculty of Biology and Agriculture, University of Rzeszów, Zelwerowicza 4 St., 35-601 Rzeszów, Poland

**Keywords:** elderberry, elderflowers, food colorant, jelly, phenolic profile, anthocyanins

## Abstract

The aim of the study was to obtain a dye from black elder fruits and flowers and to study their potential in production of jellies with high antioxidant activity. Three dyes were produced by lyophilization of aqueous extracts: (1) fruits dye (F), (2) flowers dye (FL), and (3) fruits and flowers dye (F + FL). Their polyphenol profiles were compared by means of ultra-performance liquid chromatography (UPLC). The antioxidant activity [ferric reducing/antioxidant power assay (FRAP) and DPPH radicals scavenging test and total phenolics were compared by spectrophotometric methods. Jellies were produced from agar and gelatin with the addition of three obtained dyes, and their antioxidant water- and lipid soluble fractions were tested with a Photochem device. Results indicated that black elder fruits are rich in anthocyanins, especially cyanidin-3-*O*-sambubioside (7.56 mg/g d.w.), while flowers are rich in polyphenols, especially chlorogenic acid (2.82 mg/g d.w.) and rutin (4.04 mg/g d.w.). FL dye exhibited higher antioxidant activity compared to F dye (for about 30–40%), regardless of the used method, whereas F + FL dye was characterized by intermediate antioxidant activity. Jellies produced with the addition of FL dye had better antioxidant properties but unattractive color and unpleasant taste, but the use of F + FL dye created a product of favorable organoleptic properties and antioxidant activity comparable to jellies with F dye addition.

## 1. Introduction

Black elder (*Sambucus nigra* L.) is a plant widely used in many parts of the world, often referred to as the tree of life. It grows in damp places, most often outskirts of forests, wastelands, thickets, and riversides [1,2]. Black elder fruits are a rich source of antioxidant compounds, mainly anthocyanins [3,4]. They are also an excellent source of vitamins, minerals, and carbohydrates [5]. Scientific reports indicate that the *Sambucus nigra* L. fruits can be used as a functional food in the prevention and the treatment of disorders associated with the deficiency of certain elements in the human diet. The portion of 100 g of black elder fruits allows the implementation of 20% of the recommended consumption of the main elements in the human diet [6]. Moreover, both the fruits and the flowers are sources of flavonols (e.g., kempferol, quercetin, isoramnetin, and their derivatives), proanthocyanidins, and phenolic acids (e.g., chlorogenic, neuro-chlorogenic, or crypto-chlorogenic acid) [3,4]. The rich aroma of black elder raw material is influenced by essential oils occurring in an amount of about 0.01% and containing about 53 compounds [1,7]. Meanwhile, black elder fruits and flowers are used as medicinal components rather than as food ingredients. However, they are more frequently used in the manufacture of various types of liqueurs, jams, and juices [8].

Black elder fruits are used in the treatment of psoriasis and all skin changes, thus they have found a wide application in cosmetics and the production of natural creams. Quite often, black elder is associated with anti-inflammatory, antibacterial, soothing edema, and inflammation properties, therefore it is a component of syrups for sore throat and expectorants in the treatment of mild inflammation of the upper respiratory tract [5,6,9]. Black elder fruits and flowers also have the potential to treat respiratory problems such as hay fever and asthma. Due to the high content of vitamins and polyphenols, they are often used to ward winter illnesses and strengthen immunity [10]. Further, dried fruits and flowers are used as diuretic and diaphoretic medicines [5].

Dried flowers of black elder (*Sambuci floss*) are most often used in various tea compositions, but they are also used in several cosmetics and medicinal products. It is well known that black elder flower infusions are used as cough syrups [8].

Anthocyanins found in a significant amount in black elder fruit are natural dyes qualified to the flavonoid group [11]. Obtaining the black elder dye allows the creation of many new technological products as well as the improvement of existing ones. The low cost of manufacturing, the intensity of colorizing, and the safety of its use encourages manufacturers to apply it in food technology. Another positive feature attributed to such dyes is the protection of food products against deterioration, as they antagonistically affect certain fungi, bacteria, and viruses [12]. The factor limiting technological use of black elderberry is the presence of a toxic cyanogenic glucoside compound—sambunigrin—in its extract. This disadvantage is, however, easy to avoid by blanching fruits prior to main processing [13].

The aim of the research was to obtain a natural food dye with high antioxidant properties based on black elder fruits and flowers mixture and to study its implementation in the production of hand-made jellies with high antioxidant activity.

## 2. Material and Methods

### 2.1. Raw Material

The material for the research consisted of fresh black elder (*Sambucus nigra* L.) fruits and flowers obtained in 2018 from 3 different shrubs growing in the forestry site in ecologically clean places near Lublin (Poland). Fruits were collected at the beginning of September and were preserved by two different methods: (1) blanching and then freezing at −21 °C, and (2) convective drying in a laboratory dryer at 60 °C for 5 h. The flowers were harvested at the end of May and immediately after picking were dried by convective drying in a laboratory dryer at 60 °C for 3 h. After the treatment, the flowers showed a yellow color and were separated from the stalks.

### 2.2. Preparing of Black Elder Dyes

Three black elderberry dyes: (1) from elder fruits (F); (2) from elder flowers (FL); (3) from fruits and flowers (F + FL) were obtained by the lyophilization method in the Telstar Lyo Quest shelf lyophilizer. Briefly, 100 g of frozen, previously blanched fruits, 10 g of dried flowers, as well as a mix of 100 g of fruits and 10 g of flowers were ground in a mortar with 200 mL of distilled water and transferred quantitatively to beakers. The mixture was brought to boiling on the laboratory electric cooker and kept in this state for 10 min. After that, the solutions were filtered through sterile gauzes, and the filtrates were frozen to −60 °C during 24 h and suspended to lyophilization. Dehydration was carried out for 48 h by heating the shelves to 30 °C at a normal pressure of 0.5 bar. The dehydrated material was crushed using a metal spatula, secured in wire bags, and stored at 21 °C in a dry, dark place until analyses but for no more than 2 weeks.

### 2.3. Application of Black Elderberry Dyes in Jellies Preparation

Several kinds of jellies with the addition of the obtained dyes were prepared. As fixatives, gelatin and agar were used, and as a sweetener, multifloral honey obtained directly from a beekeeper operating in Lublin region (Poland) was added. The composition of the produced jellies is presented in Table 1.

### 2.4. Analysis of Polyphenol Profile of Raw Materials Performed by UPLC Method

#### 2.4.1. Sample Preparation

The polyphenol profile was analyzed in black elder dried and frozen fruits as well as in dried flowers. Sample extracts were prepared by grinding 5 g of the sample in a mortar with 25 mL of solvent (74% ethanol with the addition of 1% formic acid). The obtained solutions were then homogenized at room temperature for 2 min using a mechanical laboratory homogenizer (OMNI TH-02, Waterbury, CT, USA) and placed for 15 min in an ultrasonic water bath (Polsonic, SONIC 10, Warsaw, Poland) without additional heating. Next, samples were centrifuged at 3500 rpm for 10 min, and the supernatant was decanted. A three-step solvent elution process for each sample was carried out for polyphenols extraction, and the total volume of the solvent used (acidified ethanol) was 75 mL. The extracts were then evaporated in a rotary evaporator Hei-VAP Advantage (Heidolph Instruments, Walpersdorfer, Schwabach, Germany) at 35 °C under reduced pressure. The samples were then redissolved in 6 mL of the solvent and, prior to application into the column, were diluted 10 times and filtered through a 0.45 μm nylon filters.

#### 2.4.2. Analysis Parameters

Testing of the polyphenol compounds profiles of the fruits (frozen and dried) as well as the dried flowers was performed by ultra-high performance liquid chromatography coupled with photo diode array detector and electrospray ionization-mass spectrometry (UPLC-PDA-ESI-MS). For this purpose, an Aquision ultra-efficient liquid chromatograph from Waters (Micromass, Manchester, UK) equipped with a diode array detector (PDA) and a tandem mass detector in the form of a double quadruple (TQD) was used. The separation was carried out on a C18 BEH column with dimensions of 100 mm × 2.1 mm and a grain size of 1.7 μm (Waters, Milford, MA, USA). The following gradient mobile phase was applied: from 20% B (40% acetonitrile) and 80% A (0.1 % of an aqueous formic acid solution) to 100% B and 0% A in 8 min. The separation was performed at a mobile phase speed of 0.35 mL/min and a column temperature of 50 °C. The analysis time was 9.5 min. The injection volume was 5 μL. The parameters of the mass detector were as follows: 3.5 kV capillary voltage, 45 V sample voltage, and the temperature of the ion source and the desolvation were 120 °C and 350 °C, respectively. Nitrogen with a flow rate of 800 l/h was used as the carrier gas. The detection was carried out in the negative ion mode in the m/z range from 120–1200. Data collection and analysis were performed using Mass-Lynx 4.1 software (Waters).

#### 2.4.3. Qualitative Analysis

The compounds were identified by a comparison of UV absorption maximum spectra, retention times, molecular weight determined on the basis of the mass-to-charge ratio, and fragmentation spectra with the available standards and information obtained from the literature. The disintegration spectra were obtained by using collision induced fragmentation (CID) in a tandem system. Collision energy was selected individually for the analyzed compound.

#### 2.4.4. Quantitative Analysis

In the quantitative analysis process, single ion recording (SIR) was used for selective ions characteristic of wanted compounds with predetermined spectra. Multiple reaction monitoring (MRM) was also used, in which the presence of fragmentation ions from the selected precursor was detected. The calculations were made on the basis of calibration curves for the dependence of the peak area on the concentration applied to the column of the compound. The calculations were made using Microsoft Excel 2010 (Microsoft Corporation, Redmond, Washington, DC, USA)).

### 2.5. Antioxidant Properties of Obtained Dyes

#### 2.5.1. Ferric Reducing/Antioxidant Power Assay

The antioxidant properties of the aqueous dyes solution were determined using a ferric reducing/antioxidant power assay (FRAP method) according to a modified version of the procedure described by Benzie and Strain [14]. Aliquots of 0.2 mL of 10 % (*w*/*v*) dye solution were mixed with 1.8 mL FRAP reagent [2.5 mL of 10 mM 2,4,6-Tris(2-pyridyl)-s-triazine (TPTZ) (Sigma Aldrich Co., Saint Louis, MO, USA) solution in 40 mM HCl, 2.5 mL of 20 mM FeCl_3_ (Sigma Aldrich Co., USA), and 25 mL of 0.3 M acetate buffer (pH 3.6)], and after 10 min incubation in 37 °C, absorbance was measured at 593 nm. A calibration curve was prepared using a Trolox standard solution at concentrations of 0–350 nmol/mL. The results were expressed as the FRAP value (µmol Trolox/g of dye).

#### 2.5.2. DPPH Radical Scavenging Activity

Radical scavenging activity (RSA) was investigated using the 2.2-diphenyl-picrylhydrazyl radical (DPPH) according to the procedure performed by Anton et al. [15]. Aliquots of 0.2 mL of 10% (*w*/*v*) dye solution were mixed with 1.8 mL metanolic DPPH solution (0.1 mM). The decrease in DPPH absorbance (*A*) was measured at 517 nm according to the blank (*A*_0_) after 60 min incubation at a temperature of 21 °C. The DPPH radical scavenging activity expressed as a percentage was calculated as:

RSA% = ([*A*_0_ − *A*]/*A*_0_) × 100%

#### 2.5.3. Total Phenolic Content

The total phenolic content (TPC) of the obtained aqueous dyes solutions was determined using the method of Singleton and Rossi [16] with minor modifications. Aliquots of 0.2 mL of 10% (*w*/*v*) dye solution were mixed with 1 mL of Folin–Ciocalteu reagent (Merck, Darmstadt, Germany), previously diluted 1:10 with distilled water, followed by the addition of 0.8 mL of 7.5% sodium carbonate (Na_2_CO_3_; POCH Gliwice, Poland). After incubation at room temperature for 120 min, the absorbance of the reaction mixture was measured at 760 nm against blank. The TPC was expressed as mg of gallic acid (Sigma Aldrich Co., USA) equivalents (GAE) per g (mg/g) of dye using the calibration curves of gallic acid prepared in the range 0–250 µg/mL.

### 2.6. PCL Assay

For the analysis of the capacity of water-soluble (ACW) and lipid-soluble (ACL) antioxidant fractions of obtained jellies, the photochemiluminescence (PCL) method using a Photochem^®^ device (Analytik Jena AG, Jena, Germany) was applied. In ACW, fractions of antioxidants such as flavonoids, ascorbic acid, aminoacids, etc., were detected, while in ACL, fractions of tocopherols, tocotrienols, carotenoids, etc., were measured [17]. The study was performed using a commercial reagent kit for ACW and ACL (Analytik Jena AG, Jena, Germany). Finely chopped jellies were crushed in a mortar with extracting solution (1:10 *w*/*v*) water for ACW and methanol for ACL. The amount of 50 μL of properly diluted extract was mixed with ready reagents (ACW or ACL) according to the attached instructions. The prepared mixture was placed in the Photochem device equipped with PCLSoft 5.1 software, which gave the final results. Results were calculated on the basis of standard curves into mmol ascorbic acids (AA) equivalents per 100 g of jellies (mmol/100 g) for ACW and μmol Trolox equivalents (TE) per 100 g of jellies (μmol/100 g) for ACL.

### 2.7. Statistical Analysis

The results are presented as the mean values with standard deviations (SD). Significant differences (*p* < 0.05) between tested parameters were determined using one-way analysis of variance followed by Tykey’s test. All calculations were obtained using Statistica 10.0 software (StatSoft, Inc., Tulsa, OK, USA).

## 3. Results and Discussion

Black elder is a plant strongly prevalent in our ecosystem and environment, and its surprising but possible health properties are being increasingly noticed. Depending on the felling process, the vegetation period, and the growing conditions, the plant may exhibit different contents of polyphenolic compounds and biological activity [12]. Therefore, it is necessary to determine the quality, i.e., the antioxidant potential of individual parts of this plant.

### 3.1. Raw Materials Comparison by UPLC

Due to the fact that black elder fruits are mainly a source of anthocyanins and only a minor fraction of phenolic acid was present, the quantities of four major anthocyanins (cyanidin-3-*O*-sambubioside-5-glucoside, cyanidin-3,5-diglucoside, cyanidin-3-*O*-sambubioside, and cyanidin-3-*O*-rutinoside) were compared in both dried and frozen fruits (Table 2). Frozen black elder fruits were characterized by a significantly higher (*p* < 0.05) total content of anthocyanins than dried fruits (by 40%). The preservation process in this case had a big impact on the content of active compounds in the raw material. For the production of the dye, only fresh or frozen fruit previously subjected to blanching are suitable.

In general, several factors are believed to affect the stability of anthocyanins in fruits during preparation, processing, and storage, including: pH, temperature, light, oxygen, metal ions, enzymes, and sugars. Moreover, the method of anthocyanins extraction prior to analysis can have key impacts on final results [18]. However, our obtained results are in agreement with the results of the study conducted by Anton et al. [15] on fresh black elder fruit, where the same four main anthocyanins were identified. They found cyanidin-3-*O*-sambubioside to be the most abundant anthocyanin (1.34 mg/g d.w.), however, their results were lower compared to the present study (Table 2). Cyaninidin 3-*O*-sambubiosie-5-glucoside was the least abundant anthocyanin for both studies. According to Wu et al. [18] and Veberic et al. [6], the two prevalent anthocyanins in *S. nigra* are cyanidin-3,5-diglucoside and cyanidin-3-*O*-sambubioside, accounting for more than half of all determined anthocyanins, which is in agreement with our investigations. 

Various researchers have examined elderberries without describing cultivars and reported the same four peaks as the samples tested in this study [6,12]. However, Wu et al. [18], for the first time, identified three additional minor anthocyanins present in *S. nigra* (cyanidin 3-rutinoside, pelargonidin 3-glucoside, and pelargonidin 3-sambubioside), a non-cyanidin-based anthocyanin. Cyanidin 3- rutinoside was also detected in the present study.

Black elder flowers are known to be a rich source of phenolic acids and flavonoids; therefore, their polyphenolic profile was tested by UPLC, and 11 different compounds were detected (Table 3). Quercetin 3-rutinoside and chlorogenic acid were found in the highest concentrations, while the remaining compounds were in marginal amounts (<1 mg/g). This is in agreement with Christensen et al. [19], who reported six flavonol glycosides and 11 phenolic acids in black elder flowers and their extracts. Among flavonoids, they identified quercetin-3-rutinoside (rutin), quercetin-3-glucoside (isoquercitrin), kaempferol-3-rutinoside, isorhamnetin-3-rutinoside, isorhamnetin-3-glucoside, and quercetin-3-6-acetylglycoside, whereas among phenolic acids, derivates of quinic acid containing caffeic or *p*-coumaric acid moieties were determined. However, they reported higher concentrations of rutin in black elder flowers (ranging between 11.5 and 42.3) depending on the genotype, as compared to the present study. Such differences could be due to the fact that the measurements were admittedly expressed as dry mass but were performed in fresh flowers samples. According to Schmitzer et al. [5], flavonol glycosides rutin, kaempferol-3-rutinoside, and isorhamnetin-3-rutinoside are the major flavonoids in black elder flowers, contributing as much as 90% to the total flavonoids content. Opposed to the chemical composition of black elder fruits, which are particularly rich in anthocyanins, flowers do not contain any pigments from this group. However, they are characterized by a greater concentration of flavonoids [11]. High content of both chlorogenic acid and rutin create black elder flowers extremely beneficial to human health. According to Gil and Wianowska [20], chlorogenic acid exhibits many biological properties, such as antioxidant, inhibition of the HIV-1 integrase, and inhibition of the mutagenicity of carcinogenic compounds. Moreover, recently, it was hypothesized that such a compound is helpful in fighting the obesity and modifying glucose-6-phosphatase involved in glucose metabolism, whereas rutin helps blood circulation, prevents blood clots, and lowers cholesterol [21]. 

### 3.2. Antioxidant Activity of Black Elder Dyes 

Based on the analysis of the profile of phenolic compounds, it was found that both black elder fruits and flowers contain different major components responsible for their biological activity. Thus, the combination of both materials should create an additive effect and result in obtaining the product characterized by enriched chemical composition. Dyes obtained from fruits and flowers were tested for the level of antioxidant activity by FRAP and DPPH tests as well as the total content of phenolic compounds with Folin–Ciocalteu reagent. Results indicated that, regardless of the method used, the dye obtained from fruits presented lower antioxidant activity compared to the dye prepared from flowers (Table 4). Similar results were obtained by Kołodziej and Drożdżal [22]. They also detected greater antioxidant activity in flowers compared to black elder fruits, and the average values for FRAP were 0.39 and 0.27 mmol Fe^2+^/g d.m. respectively. The present study results are also in accordance with the outcome of Dawidowicz et al. [23], who stated that black elder flowers exhibited much stronger neutralizing activity of free radicals measured by the DPPH test compared to black elder fruit (95.15 and 67.69%, respectively). Anton et al. [15] reported the percentage of inhibition of free radicals by DPPH tests in black elder fruits at the level of 63.26%, comparable to results in the present study (68.23%).

Various components such as phenolic acids, flavonols, and anthocyanins are responsible for the antioxidant properties of black elder fruits and flowers. The black elder fruits and flowers mix dye exhibited significantly (*p* < 0.05) higher levels of phenolic compounds (by 25%) as compared to fruit dye. This indicated that the flower dye evidently enriched the fruit dye with phenolic compounds. Such a combination led us to obtain a product with a nice purple color and strong antioxidant properties. These results are supported by the findings of Kołodziej and Drożdżal [22], who also confirmed a higher content of polyphenols in flowers as compared to fruits. Research carried out by Vapiana and Wesolowski [23] also indicated a higher content of phenolic compounds in flowers than in fruits (19.81–23.90 mg GAE/g d.w. raw material vs. 15.23–35.57 mg GAE/g d.w. raw material, respectively). This indicates that flowers and fruits are both rich in polyphenolic compounds, and that it is worth combining them together to create, for example, dyes. Moreover, dye from fruits has a pleasant red color but a lower content of antioxidants, while dye from flowers shows strong antioxidant potential but a pale yellow color. For this reason, we decided to obtain a new dye by combining the black elder fruits and flowers together.

Statistical analysis showed strong positive Pearson’s correlation between total phenolic compounds and DPPH tests (*r* = 0.936) as well as FRAP tests (*r* = 0.957), indicating that polyphenols were strongly responsible for the antioxidant activity of the tested dyes. Such an observation is in agreement with the studies of Anton et al. [15], who found a strong positive correlation between DPPH tests and anthocyanins content, which belong to phenolic compounds. 

### 3.3. The Effect of Black Elder Dye on Antioxidant Activity of Jellies

High antioxidant activity and effective coloring strength of black elder dyes have led to their use in the production of jellies. As a control, samples sweets without dyes were prepared. Jellies made on the basis of agar and gelatin were characterized by better textural features, e.g., toughness and chewiness, as compared to the jellies made of gelatin alone. In terms of organoleptic properties, jellies with the addition of mixed fruits and flowers dye were characterized by the most attractive purple color as compared to jellies with dye from the flowers (which gave a brown color) and with the fruits dye, which gave a dark red, almost black color of jellies that would not be encouraging for children. Due to the fact that the obtained dyes showed various antioxidant activity, the antioxidant power of final product was tested. The content of water-soluble (PCL-ACW) and fat-soluble (PCL-ACL) antioxidants in the produced jellies with dyes from black elder fruits, flowers, and a combination of them was determined by the photochemiluminescence method (Table 5). As a control sample, two types of jellies were made: gelatin-based and jellies prepared from a combination of gelatin and agar. The highest content of both water- and fat-soluble antioxidant fractions revealed the jellies with the addition of flowers dye. Jellies obtained from the combination of black elder flowers and fruits showed only slightly lower antioxidant activity than jellies with the addition of flowers dye and a significantly higher antioxidant performance than jellies with fruits dye. Moreover, the results indicated that, regardless of the applied dye, the water-soluble fraction had significantly higher antioxidant capacity than the fat-soluble one. 

Analyzing the effect of the fixer on the antioxidant properties of jelly bears, it was found that the use of a combination of gelatin and agar insignificantly improved the antioxidant properties of jellies, especially in the water-soluble fraction, while gelatin alone increased the level of the lipid-soluble antioxidant fraction. Therefore, the ACW/ACL ratio was higher in the samples with the addition of both fixers. This could have been due to the fact that agar is of plant origin produced from seaweeds, which are rich in phenolic compounds, while gelatin is of animal origin and contains proteins and peptides [24].

Jellies with the addition of black elder flower dye showed the highest antioxidant activity; however, their color was not attractive. The production of jellies with an addition of a mixture of dyes from black elder fruits and flowers allowed us to obtain a product with an attractive purple color, comparable to a commercial product in terms of chewiness with organoleptic properties as taste, appearance, and smell, as well as with high antioxidant properties.

## 4. Conclusions

Results indicate that frozen black elder fruits have a higher content of anthocyanins as compared to dried fruits by about 81% with a predominant presence of cyanidin-3-*O*-sambubioside in frozen and cyanidin-3-*O*-sambubioside-5-glucoside in dried fruits. The UPLC analysis of black elder flowers confirmed the presence of 11 different polyphenols with the dominance of quercetin 3-rutinoside and chlorogenic acid. The process of obtaining black elder dye from frozen fruits and flowers leads to the creation of a new technological product of natural origins, which is characterized by an attractive purple color and is thus a good alternative to the commercially available chemical pigments. The dye obtained from the combination of black elder fruits and flowers showed a 30% higher level of antioxidant activity and a 25% higher level of phenolic compounds as compared to dye from fruits. The procedure of the addition of a new dye (the mix of elder fruits and flowers) to jellies produced during research raised the level of water-soluble antioxidant fraction by 27% and for fat-soluble antioxidants by 25% as compared to jellies with the addition of fruit dye.

## Figures and Tables

**Table 1:** Composition of produced jellies.

No. of Sample	Gelatin Content [g]	Agar Content [g]	Water Content [mL]	Honey Content [g]	Dye Content [1 g]
1	3	-	51	5	-
2	3	-	50	5	F
3	3	-	50	5	FL
4	3	-	50	5	F + FL
5	1.5	1.5	51	5	-
6	1.5	1.5	50	5	F
7	1.5	1.5	50	5	FL
8	1.5	1.5	50	5	F + FL

In brief, gelatin was dissolved in hot water (90 °C); after cooling to 40–50 °C, honey was dissolved, and dyes were added. The whole mixture was mixed carefully but well with a glass baguette to prevent the formation of air bubbles. Then, the mixture was transferred into silicone molds with the shape of small bears and left in the fridge (4 °C) for one hour. As a control, sample jellies without dyes were used.

**Table 2:** Identified anthocyanins and their quantities in the frozen and dried back elder fruits.

Identified Anthocyanins	*m/z*	R_t_	Black Elder Frozen Fruit [mg/g d.w.]	Black Elder Dried Fruit [mg/g d.w.]
Cyanidin-3-*O*-sambubioside-5-glucoside	743	2.26	0.04 ± 0.00 ^b^	1.02 ± 0.15 ^a^
Cyanidin-3,5-diglucoside	581	2.86	1.10 ± 0.05 ^a^	0.33 ± 0.02 ^b^
Cyanidin-3-*O*-sambubioside	449	2.97	1.97 ± 0.02 ^a^	0.27 ± 0.03 ^b^
Cyanidin-3-*O*-rutinoside	611	4.55	0.10 ± 0.00 ^b^	0.63 ± 0.07 ^b^
**Total anthocyanins**			**3.21**	**2.25**

*m/z*- mass-to-load ratio; R_t_- retention time; ^a, b^ significant differences (*p* < 0.05) in a column.

**Table 3:** The content of individual polyphenols identified in black elder flowers by ultra-performance liquid chromatography (UPLC).

Identified Compound	*m/z*	R_t_	Result [mg/g d.w.]
Neochlorogenic acid	353	2.357	0.77 ± 0.09 ^b^
Chlorogenic acid	353	2.983	2.82 ± 0.35 ^a^
Cryptochlorogenic acid	353	3.127	0.21 ± 0.03 ^b^
Coumaroyl-quinic acid	337	3.681	0.39 ± 0.05 ^b^
Quercetin di-glucoside	625	3.977	0.18 ± 0.03 ^b^
Quercetin 3-rutinoside	609	4.594	4.04 ± 0.57 ^a^
Quercetin 3-glucoside	463	4.806	0.56 ± 0.06 ^b^
Kaempferol 3-rutinoside	593	5.154	0.13 ± 0.01 ^b^
Glucuronide-rhamnoside quercetin	623	5.299	0.95 ± 0.08 ^b^
Kaempferol 3-rutinoside	447	5.364	0.25 ± 0.02 ^b^
Quercetin glucuronide	477	5.538	0.26 ± 0.03 ^b^

*m/z*- mass-to-load ratio; R_t_- retention time; ^a, b^ significant differences (*p* < 0.05) in a column.

**Table 4:** Results of the analysis of antioxidant properties of the tested dyes.

Sample	FRAP [µmol TE/g]	DPPH [% inhibition]	TPC [mg GAE/g]
F dye	597.46 ± 17.97 ^b^	68.23 ± 2.24 ^b^	14.68 ± 2.09 ^b^
FL dye	947.98 ± 21.75 ^a^	96.24 ± 4.84 ^a^	25.34 ± 5.41 ^a^
F + FL dye	849.58 ± 23.96 ^a^	90.11 ± 3.47 ^a^	19.58 ± 7.58 ^b^

^a, b^ significant differences (*p* < 0.05) in a column. FRAP- ferric reducing/antioxidant power assay; TPC- total phenolic content; GAE- gallic acid equivalents.

**Table 5:** The content of hydrophilic and hydrophobic antioxidants in tested jellies [photochemiluminescence (PCL) method].

Used Additive.	PCL-ACW [µmol AA/1g Product]	PCL-ACL [nmol Trolox/1g Product]	ACW/ACL Ratio
Gelatin version			
Control	1.49 ± 0.34 ^a^	4.22 ± 0.37 ^a^	0.35
F dye	25.75 ± 1.23 ^b^	5.12 ± 0.03	5.03
FL dye	55.75 ± 0.38 ^c^	7.69 ± 0.11 ^b^	7.25
F + FL dye	43.05 ± 0.68 ^c^	7.08 ± 0.09	6.08
Gelatin-agar version			
Control	2.34 ± 1.53 ^a^	3.97 ± 0.34	0.59
F dye	29.21 ± 1.85 ^b^	4.93 ± 0.16	5.92
FL dye	58.38 ± 1.90 ^c^	7.19 ± 0.02	8.11
F + FL dye	48.50 ± 1.30 ^c^	6.52 ± 0.08	7.43

^a, b, c^ significant differences (*p* < 0.05) in a column within the group of gelatin and gelatin-agar version. ACW- water-soluble; ACL- lipid-soluble.
